# *hafoe*: an interactive tool for the analysis of chimeric AAV libraries after random mutagenesis

**DOI:** 10.1038/s41434-025-00548-3

**Published:** 2025-07-08

**Authors:** Tatevik Jalatyan, Erik Aznauryan, Rokib Hasan, Valeri Vardanyan, Stepan Nersisyan, David B. Thompson, Noah Davidsohn, Sanya Thomas, Simon van Haren, Jenny Tam, Denitsa Milanova, George M. Church, Lilit Nersisyan

**Affiliations:** 1 Armenian Bioinformatics Institute, Yerevan, Armenia; 2 Institute of Molecular Biology, National Academy of Sciences of Armenia, Yerevan, Armenia; 3 Wyss Institute, Harvard University, Boston, MA USA; 4 Harvard Medical School, Harvard University, Boston, MA USA; 5 Rejuvenate Bio, San Diego, CA USA; 6 Kavli Institute for the Physics and Mathematics of the Universe, The University of Tokyo, Tokyo, Japan; 7 Precision Vaccines Program, Department of Pediatrics, Boston Children’s Hospital, Boston, MA USA; 8 Present Address: Computational Medicine Center, Thomas Jefferson University, Philadelphia, USA

**Keywords:** Genetic vectors, Genetic vectors

## Abstract

Naturally occurring adeno-associated viruses (AAVs) are an integral part of gene therapy, yet engineering novel AAV variants is necessary to expand targetable tissues and treatable diseases. Directed evolution, particularly through DNA shuffling of the capsid genes of wild-type AAV serotypes, is a widely employed strategy to generate novel chimeric variants with desired properties. Yet, the computational analysis of such chimeric sequences presents challenges. We introduce *hafoe*, a novel computational tool designed for the exploratory analysis of chimeric AAV libraries, which does not require extensive bioinformatics expertise. *hafoe* accurately deciphers the serotype composition and enrichment patterns of chimeric AAV variants across different tissues. Validation against synthetic datasets demonstrates that *hafoe* identifies parental serotype compositions with an accuracy of 96.3% to 97.5%. Additionally, we engineered chimeric AAV capsid libraries and screened novel AAV variants for tropism to human dermal fibroblasts and dendritic cells, as well as canine muscle, and liver tissues. Using *hafoe* we identified and characterized enriched AAV variants in these tissues for potential use in gene therapy and vaccine development. *Overall, hafoe* can provide valuable insights that may further support the rational design of AAV vectors based on parental serotype and sequence preferences of the capsid genes in target tissues.

## Introduction

Targeted delivery of genes to the tissue of interest is one of the most challenging aspects of gene therapy [1, 2]. Viral vectors, such as retroviruses, adenoviruses, adeno-associated viruses (AAV), and herpes simplex viruses are promising options due to the diversity of cell attachment and entry mechanisms [1, 3]. AAV vectors have become the most popular choice among other viral vectors, due to lower immunogenicity, higher safety profiles, ease of production, and robust expression of the transgene in various tissue types [2, 4]. Recombinant AAV vectors (rAAV) have demonstrated success in preclinical and clinical trials for various gene replacement, gene silencing, and gene editing applications [5]. Several rAAV therapies have already obtained regulatory approval in either Europe or the United States [5–8]. Despite these successes, there is a need to increase their efficiency of optimal interactions with target tissue receptors and better immunological tolerance [1, 4, 5].

AAVs are non-enveloped viruses with a protein capsid surrounding a 4.7 kilobase-long single-stranded DNA. The AAV genome encodes non-structural (*rep*), structural (*cap*), assembly activating (*aap*), and membrane-associated accessory (*maap*) proteins [9]. The *cap* gene encodes three virion proteins (VPs): VP1, VP2, and VP3 that assemble into a 60-subunit capsid. VP3 is the most prevalent capsid protein, accounting for approximately 50 of the 60 capsid monomers [10, 11]. VP3 contains a highly conserved core region and nine distinct variable regions (VRs), which are associated with functional roles in the AAV life cycle essential for successful gene delivery, including receptor binding, tissue transduction, and antigenic specificity [12].

More than 12 wild-type AAV serotypes have been studied as gene delivery vectors and several used in clinical trials [4, 13]. These serotypes have different binding receptors and different tissue tropism profiles in terms of transduction efficiency [1, 4]. However, wild-type AAV vectors still suffer from broad tissue tropism, which can result in non-specific delivery to unintended tissues, low transduction efficiency, and undesired immune responses [1, 4, 5]. Hence, numerous strategies are utilized to engineer novel capsid variants to decrease the viral load and improve the target delivery and immune system evasion properties [1, 4].

When the biology of the receptor binding at a specific tissue is known, rational capsid engineering can be performed via structure-guided evolution, peptide or protein insertions, chemical conjugation [2, 14]. A more robust approach to developing AAV capsid variants to an unknown tissue target is directed evolution, which subjects the capsid genes to iterative rounds of mutagenesis and selection. DNA shuffling is one of the widely used directed evolution techniques for creating a library of chimeric variants by random fragmentation of the *cap* gene of naturally occurring AAV serotypes and homologous recombination-based reassembly of the fragments using primerless PCR. The subsequent step is the iterative selection of the resulting AAV variants by applying them to target cells and isolating the enriched variants [15–19].

The most compelling advantage of directed evolution methods is the large volume of positive as well as negative data that can inform subsequent protein designs. However, data analysis of AAV and other chimeric DNA/protein libraries is challenging and not straightforward. Among the tools that have been developed to aid researchers in the analysis of these experiments are Xover [20], Shuffled [21], Parent-map [22] and ShuffleAnalyzer [23]. However, those are based on multiple sequence alignment or comparison of each variant sequence against all parental sequences to find exactly matching fragments and can take only a limited number of variant vectors as input. In addition, these tools offer limited output which at times may not be sufficient for complete analysis of parental recombination and subsequent mutation events [20, 21, 24].

Here, we present a new computational tool, *hafoe*, a command-line-based stand-alone tool to facilitate the automated exploratory analysis of AAV chimeric libraries and the identification of enriched variants in desired tissues. The tool is easy to use for non-bioinformaticians. In contrast to existing tools, *hafoe* can rapidly process hundreds of thousands of variants at once, clustering and summarizing the results with interactive graphical reports that aid in the analysis of recombination patterns of the chimeric vectors. We validate its performance using in silico*-*generated as well as published datasets. We also used it to quantify and characterize chimeric vectors enriched in human fibroblasts and dendritic cells as well as canine muscle and liver tissues, leading to the identification of novel AAV capsids for potential gene therapy and vaccine development applications.

## Materials & methods

### Analysis pipeline

**Fig. 1:**
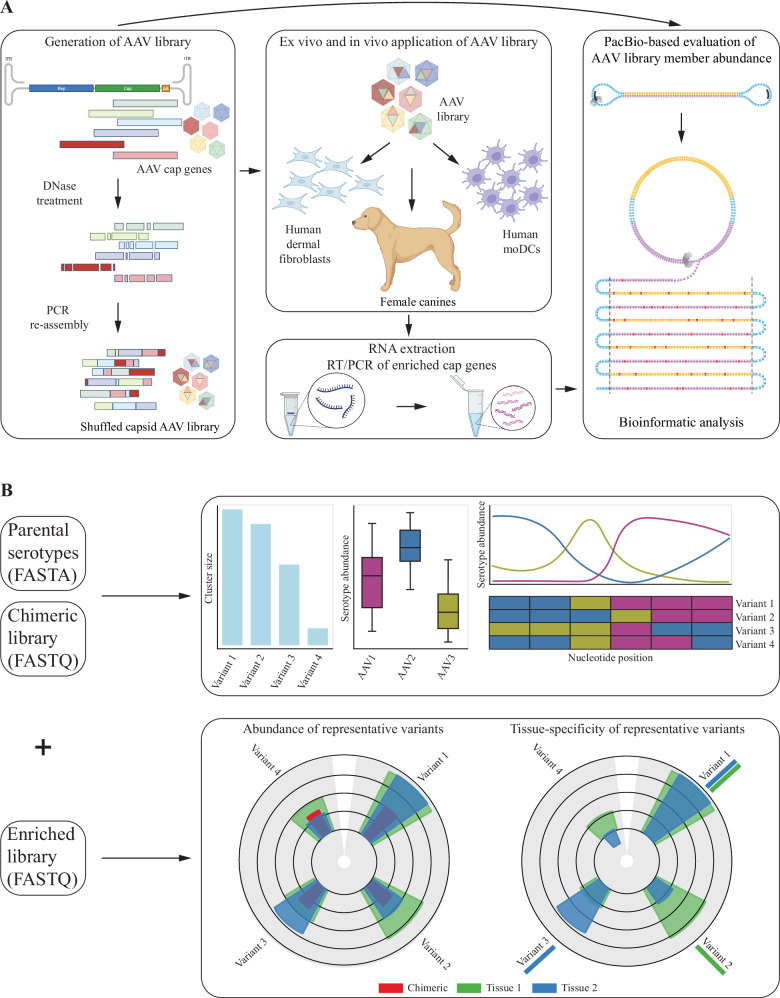
Schematic overview of experimental design and workflow of *hafoe.* **A** Experimental design for generating chimeric AAV capsid library from parental AAV serotypes, followed by enrichment in DCs and HDFs, total RNA extraction, and PacBio sequencing. **B** *hafoe* takes as input parental AAV serotypes and libraries of chimeric variants before and/or after enrichment in target tissues. *hafoe* produces interactive graphics representing the identified clusters of similar chimeric vectors, showing the prevalence of each wild-type vector and serotype composition of chimeric representatives. It also shows the enrichment of representative sequences in target tissues and the tissue-specificity of each vector.

The *hafoe* program takes as input two files: a FASTQ or a CSV formatted file containing PacBio circular consensus sequences (CCS) and abundances of the chimeric DNA sequences and a FASTA file containing the *cap* gene sequences of the parental AAV serotypes. Assembled AAV sequences generated from other long-read sequencing platforms, such as Oxford Nanopore, can also be supplied in the same formats. Optionally, it can also take one or more additional files of FASTQ format containing the sequences of selected variants in the enriched libraries. *hafoe* can take as input multiple input libraries describing the enriched chimeric variants in different target tissues. In addition to text files, *hafoe* also produces interactive graphics in HMTL reports (Fig. 1B).

#### Preprocessing

**Fig. 2:**
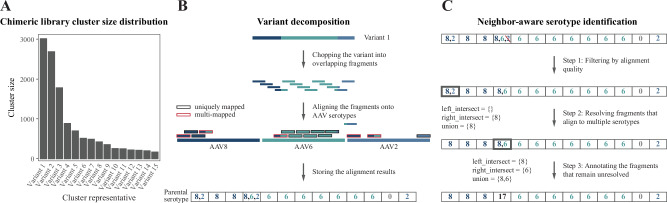
*hafoe’s* neighbor-aware serotype identification algorithm. **A** Chimeric library cluster size distribution. *hafoe* clusters the chimeric library sequences based on sequence identity and uses the representative variants of the clusters in subsequent analysis. **B** To identify the variant composition in terms of the AAV serotypes, *hafoe* first performs variant decomposition: chopping the variant into overlapping fragments of fixed size, aligning the fragments against AAV serotypes, and storing the alignment results in a list. **C** *hafoe* then performs neighbor-aware serotype identification with the following steps: for each position covered by fragments with multiple assignments Step 1) perform quality filtering (see methods), Step 2), identify the set of serotypes shared with its left and right neighbors if those exist (left_intersect and right_intersect), and update the position accordingly, Steps 3) annotate the unresolved positions as multimappers (e.g. with 17).

Both chimeric and enriched library files are filtered by sequence length and the open reading frame (ORF) boundaries. The ORFs are identified as the longest sequence regions starting from the start codon ATG and ending with either of the three stop codons (TAA, TAG, TGA) in six possible reading frames. Only variants with ORF size greater than 1.8 kb and total length less than 3 kb are selected for downstream analysis. To reduce sequence redundancy in the resulting chimeric library that arises as a result of point mutations or sequencing errors, we cluster the library members using the CD-HIT-EST program of the CD-HIT package version v4.8.1 [25] (Fig. 2A). The program performs clustering by first sorting the input sequences by length; the longest one becomes the representative of the first cluster, the remaining sequences are compared with it and if the similarity is above the selected threshold (-c 0.9), the sequences are grouped into that cluster; otherwise, the dissimilar sequence becomes a representative of a new cluster. For each comparison, CD-HIT applies a minimum number (-n 9) of identical short substrings of a given length shared by two sequences [25]. If the number of such substrings is below the set threshold, an actual sequence alignment is performed. As a similarity measure, the local sequence identity (-G 0) between two sequences is calculated by dividing the number of identical bases in alignment by the alignment length, where the alignment coverage for the longer sequence is specified as (-aL 0.9). As per our specifications, each sequence is assigned to the cluster of the highest similarity (-g 1). After clustering, *hafoe* re-assigns cluster representatives as the most abundant sequences in the chimeric library.

#### Neighbor-aware serotype identification

Neighbor-aware serotype identification is a method we have designed to describe the variant sequences and identify the variants’ compositions in terms of parental AAV serotypes. For each input sequence, we output a list of parental serotypes from which their different fragments potentially originate.

First, the variants’ sequences are chopped into overlapping fragments of equal size. By default, the fragment length is set to 100 bp, and the length of the region between the starting positions of two consecutive fragments is set to 10 bp. These default values are chosen based on the most optimal performance during our validation experiments with in silico datasets (see the text below and Supplementary Fig. 1). The fragments are then aligned to AAV serotype genomes using the Bowtie 2 program version v2.4.2 [26] (with options -a --no-unal -L 30 -D 2). The *bowtie2* command is used with increased seed length (-L 30) and reduced number of seed extension failed attempts (-D 2) options to perform a strict alignment. For each variant, the serotypes its fragments align to are stored in a list (Fig. 2B).

This list is then used for neighbor-aware serotype identification, which assigns the most probable serotype to each fragment based on its neighborhood, assuming that neighboring fragments are more probable to originate from the same serotype (Fig. 2C). The workflow proceeds as follows:

1. For each nucleotide position of the variant sequence covered by fragments with multiple serotype assignments:

1.1 we select the assignment with the maximum sum of alignment quality scores across corresponding fragments (see Supplementary Fig. 1 for the reasoning behind the selection of this metric). In case of ties, we keep all the assignments.
1.2 we identify the sets of serotypes shared with its left and right neighbors (*left_intersect* and *right_intersect* in Fig. 2C) if those exist. If a position doesn’t have a left or right neighbor, we add one with an empty serotype set assignment.
1.3 we assign to that position the union of serotypes in the *left_intersect* and the *right_intersect* sets if the union is not an empty set and leave the assignment unchanged otherwise.

2. If, after these steps, there are still unresolved positions with multiple serotype assignments, we annotate them as multi-mappers.

In the end, for each variant, *hafoe* outputs position-based assignments of parental serotypes or marks positions with multiple possible serotypes of origin.

With the default length of the region between the starting positions of two consecutive fragments (10 bp), *hafoe* detects crossover events at a resolution of 10 nt rather than determining the exact nucleotide position. To obtain nucleotide-resolved crossover positions the user can set this parameter to 1 bp.

#### Identification of enriched variants

After the assignment of serotype composition to each representative variant in the chimeric library, we wanted to explore which of those variants were successfully assembled, propagated, and enriched in the follow-up in vivo experiments in target tissues. For this purpose, *hafoe* uses the CD-HIT-EST-2D program of the CD-HIT package version v4.8.1 (with the options -g 1 -d 100 -c 0.95 -n 10 -G 0 -aL 0.95) to identify the sequences in the enriched library that are similar to the representative sequences in the chimeric library at 95% identity threshold. The total number of these sequences indicates the representative’s abundance in the enriched library. The fold change of a representative’s normalized abundance in the enriched library compared to its normalized abundance in the chimeric library was used as a measure of its enrichment. The representative variants with a log_2_ fold change greater than 1 were considered enriched, and those with a log_2_ fold change less than 1 were the reduced ones.

#### Library size normalization

The percentages of each representative variant both in the chimeric and enriched libraries were used to normalize for library size. In the case of multiple enriched libraries provided as an input, *hafoe* performs normalization of variant abundances in chimeric and enriched libraries with the median-of-ratios method suitable for between-sample comparisons [27].

#### Analysis of peptide sequences

For the analysis of these sequences, we employed the Xover tool’s algorithm (available at: http://qpmf.rx.umaryland.edu/xover.html) [20]. In brief, the Xover algorithm takes chimeric and parental protein sequences as input, performs multiple sequence alignment, and then, for each alignment position, compares the residue of the chimeric protein with the residues in parental sequences. For each position, the algorithm calculates the probabilities of the chimera’s residue originating from a given parent. Suppose there is a match between the chimera and a parental base at a given position. In that case, the algorithm evaluates the probability of the chimera’s residue originating from that parent as 1 divided by the total number of parental bases matching the chimera’s base at that position Otherwise, if there is no match, the probability is 0.

#### Software architecture

*hafoe* is written in R 4.1.3 and Bash 5.1.4 and can be executed in Unix operating systems.

ORFik (v1.12.13), microseq (v2.1.4), and seqinr (v4.2.8) are the main R packages used in the program to filter the sequences, extract open reading frame (ORF) sequences, read and write files in FASTA and FASTQ formats, and read alignment files.

The Python bokeh package (v2.4.3) is used to visualize the obtained results and export them to HTML formatted report files. The general wrapper managing the overall workflow is written in Bash 5.1.4.

#### Accuracy of variant composition assessment

To benchmark the different parameters of our algorithm, we have used different combinations of read length (the length of the chopped fragments from variant sequence), step size (the length of the region between the starting positions of two consecutive fragments), and filtering criterion (sum or average of alignment quality scores across all reads mapped to a position per serotype assignment (Fig. 2C, Step 1)) input parameters to run *hafoe* on five synthetic datasets. The accuracy of variant composition identification by *hafoe* was computed by dividing the total number of accurately described nucleotides in a sequence by the sequence length, and then averaging this value across all the sequences in the dataset (Supplementary Fig. 1).

#### Sequence conservation levels of AAV serotypes

To assess the conservation levels of parental AAV serotypes among groups of AAV variants, we first performed multiple sequence alignment of the variants of interest, using the Clustal Omega program (v1.2.4) (with the options specifying the output file format as –outfmt clu –force) [28]. Then a conservation score was derived for each position within the alignment by dividing the count of the most frequently occurring nucleotide at a given position by the total number of query sequences present in the multiple sequence alignment.

### Experimental datasets

#### Chimeric AAV capsid Library generation & virus production

To generate the chimeric AAV capsid library, capsid (*cap*) genes from 16 wildtype AAV serotypes (AAV1-13, AAVrh8, AAVrh10, AAVrh32) were PCR-amplified and mixed equimolarly. These were fragmented using DNase I, yielding 200-400 base pair fragments, which were reassembled in primer-free cycles of denaturation, annealing, and extension (Fig. 1A). The shuffled chimeric AAV capsids were then PCR amplified using a BsaI overhang containing internal primer set, digested with BsaI, and ligated into an AAV2 Rep-containing ITR-backbone vector under a strong constitutively expressing ubiquitous EF1-alpha promoter. Ligation mixtures were then transformed into bacterial competent cells. The final chimeric library plasmids were then purified, and their size and diversity were validated using PacBio sequencing. For viral particle production of the shuffled chimeric AAV libraries, a triple-transfection protocol was used including an adenoviral helper plasmid (pHelper, Aldevron), Rep plasmid (pRep) and the above-made replication-competent shuffled AAV library using HEK293T cells by SignaGen (Fredrick, MD). Specifically, a 10-layer stack with 800ug of Helper plasmid, 800 ug of Rep plasmid, and 10ug of the ITR plasmid containing capsid library was used. Capsid sequences of successfully manufactured AAV variants were PCR amplified, recloned into the ITR vector and subjected to the second AAV production as described above. This final production yielded 3 mL of virus at 2.30E12 VG/mL. The production was scaled up to obtain 10 ml of 5E13vg/ml for the animal experiments.

#### Enrichment in dendritic cells and human fibroblasts

Peripheral blood samples from healthy young adults were collected after obtaining written informed consent per protocol approved by the Institutional Review Board of Boston Children’s Hospital. De-identified, heparinized blood samples were processed within 2–4 h to isolate mononuclear (PBMCs) from the whole blood using Ficoll gradient, followed by monocyte purification using CD14+ beads (Miltenyi, 130-042-401) according to manufacturer’s protocol. Monocytes were cultured in Cellgro DC medium (Cellgenix GMBH, 20801-0500) with 250 IU/mL IL-4 ((Miltenyi, 130-093-922), and 800 IU/mL GM-CSF (Miltenyi, 130-093-866), and incubated in a 6-well plate at 37 °C and 5% CO_2_ for 5 days to generate Mo-DCs. On day 5, 2 × 106 non-adherent Mo-DCs were transferred to a new 6-well plate and 2 × 1010 library viral particles were applied. Total RNA was extracted from Mo-DCs 36 h later using RNeasy Kit. Earlier extraction time point was used to avoid biases due to differences in expression efficiency of library members (Qiagen, #74004) (Fig. 1A). Even though the variants are transcribed under the same constitutive promoter, such differences in expression could stem from codon optimality and GC content bias, as shown previously [29, 30].

Adult dermal fibroblasts were purchased from Millipore Sigma (106-05 A) and cultured in fibroblast growth medium (Millipore Sigma, 116-500) at 37 °C and 5% CO_2_. Once outgrown, 2 × 10^6 cells were infected with 2 × 10^10 library viral particles. Total RNA was extracted from dermal fibroblasts the next day using RNeasy Kit (Qiagen, #74004) (Fig. 1A).

#### Animal experiments

Canine (dog) experiments were performed at Absorption Systems California, LLC, (San Diego) labs (ASC protocol). The animal procedures were conducted in accordance with the guidelines of the National Institutes of Health (NIH) for the care and use of laboratory animals and were approved by the Institutional Animal Care and Use Committee (IACUC) at Absorption Systems. The institution is accredited by the Association for Assessment and Accreditation of Laboratory Animal Care (AAALAC) and maintains a current Public Health Service (PHS) Animal Welfare Assurance. A 1-year-old male Canine (Beagle) was injected with the AAV capsid library viruses described above; with 5E13vg/kg via intravenous route. Four weeks after injection, the Beagle was euthanized and various tissues including heart muscles, liver, and brain were harvested & flash-frozen for tissue-specific chimeric capsid enrichment and further analysis. Total RNA was extracted from the liver and muscle tissues using RNeasy Kit (Qiagen, #74004) and RNeasy Fibrous Tissue Kit (Qiagen, #74704) respectively.

#### Enriched AAV capsid recovery from liver and muscle tissues, DCs, and fibroblasts

cDNA synthesis was performed using SuperScript IV reverse transcriptase (Thermo Fisher, 18090050) and with a pool of custom-designed 10-mer sequence-specific primers (Supplementary Table 1). To increase the stability of the long cDNAs ( ~ 2230 bp), we proceeded with second-strand synthesis (Invitrogen, #A48570). Capsid variants expressed mRNA sequences were amplified from the second strand cDNA samples, with inversely oriented BasI overhang containing primers binding to upstream FwdUniversalCapsid-BsaI (5’-gggtctcgATGATTTAAATCAGGTATG -3’) and downstream RevCap_V8_BsaI primer (5’-CCATACCACATTTGTAGAGGTTT-3’) using the Platinum™ SuperFi™ PCR Master Mix (Invitrogen, #12358010). PCR amplified full capsid sequences were column purified (Qiagen, # 28104) and digested with BsaI and ligated into RJB p495 vector backbone, transformed into bacteria for plasmid amplification and later digested with HindIII (digests the backbone containing the capsids sequences with some extra sequence pads for flexibility & to improve sequence quality) and gel purified the enriched capsid pool for PacBio Sequencing.

#### PacBio Sequencing and data generation

Both the initial pooled chimeric virus samples and tissue-specific (Liver & Muscle) enriched capsid samples were used for PacBio long-read sequencing by Azenta, USA, and Histogenetics, USA respectively.

AAV sample preparation and sequencing were done by Yale Center for Genomic Analysis according to PacBio’s SMRTbell prep kit 3.0. Briefly, 7e11 vector genomes were treated with 20 U of DNase I (NEB M0303S) in a 200 μL vol for 10 min at 37 °C. DNA was extracted by using PureLinkTM Viral RNA/DNA Mini Kit (Thermo Fisher Scientific 12280050) following the manufacturer’s instructions. Samples have undergone a thermal annealing step by heating in annealing buffer (25 mM NaCl, 10 mM Tris-HCl [pH 8.5], 0.5 mM EDTA [pH 8]) at 95 °C for 5 min and then cooling to 25 °C (1 min for every −1 °C) on a thermocycler (Eppendorf Mastercycler). Libraries for vector DNA were constructed using the SMRTbell prep kit 3.0 by first performing end repair and A-tailing at 37 °C for 30 min followed by 65 °C for 5 min and subsequent addition of 4 μL of adapter per sample and Incubation at 20 °C for 30 min. Libraries were pooled and purified using 1.3X SMRTbell cleanup beads. Samples were then incubated with 5 ul of Nuclease for 15 min at 37 °C and purified again using 1.3X SMRTbell cleanup beads. Libraries were evaluated by Qubit dsDNA HS (Thermo Fisher Scientific Q32851) assay kit as well as using a DNA 7500 Bioanalyzer kit (Agilent 5067-1506). Sequencing was performed on a Sequel II instrument following standard procedures defined by the manufacturer and the Yale Center for Genomic Analysis.

### Synthetic datasets

For validation of the algorithm, we produced synthetic data of chimeric variants, simulating random fragmentation, homologous recombination, and PacBio sequencing events. First, we performed multiple sequence alignment of the 16 parental AAV serotypes, including AAV1-13, AAVrh8, AAVrh10, and AAVrh32, using the Clustal Omega program (v1.2.4) (with the options --outfmt clu --force) [28]. This information on the regions of homology was then used to simulate the DNA shuffling process. Consecutive fragments produced based on randomly chosen boundary positions in randomly chosen serotypes were concatenated to produce the chimeric variants. As PCR biases, partial digests, or varying homologous recombination and packaging efficiencies can favor certain serotypes, we assigned different probabilities to the serotype selection to mimic real-world settings as follows: AAV2 (25%), AAVrh8 (20%), AAV6 (17%), AAV7 (10%), AAV9 (7%), AAV8 (6%), AAV13 (6%), AAV1 (5%), AAV5 (2%), AAV3 (1%), AAVrh10 (1%), AAVrh32 (0%), AAV4 (0%), AAV10 (0%), AAV11 (0%), and AAV12 (0%). The fragment lengths were chosen from a uniform distribution defined within the 100-700 bp range, to reflect the usual size selection during the experimental workflows, as larger fragments can reduce diversity and shorter fragments are less prone to proper annealing [16, 31]. The information about parental compositions of the derived chimeric sequences was stored as ground truth for measuring the accuracy of our algorithm (Supplementary Fig. 1).

This method was used to generate 300 distinct chimeric sequences. Each sequence was assigned an abundance value to mimic the redundancy of sequences in the library of chimeric variants resembling the experimental settings. Specifically, 80% of chimeric sequences were randomly assigned an abundance value of 1. For the remaining 20% of chimeric sequences, abundance values were sampled from a normal distribution with a mean of 1 and a standard deviation of 50, while disregarding any values below 1.5. The SimLoRD program (v1.0.4) [32] was used to simulate PacBio circular consensus sequences (CCS) by taking each generated chimeric sequence as a reference (-rr) and its abundance count as the number of simulated reads (-n). The entire data simulation process was replicated five times to generate five different datasets using different seed values for the R functions involving random sampling.

### Published AAV vector data

The amino acid sequences of AAV vectors NP59 [33], 10A1-KP1 [34], Patent Number WO2019191701A1], and AAV-SYD12 [14] were obtained to explore parental AAV serotype contributions within these vectors.

## Results

### Identification of serotype composition and accuracy

We have developed a command-line standalone tool, *hafoe*, to provide a convenient toolbox for researchers to analyze shuffled libraries of chimeric AAV vectors.

The user provides *hafoe* with a FASTA file containing parental serotype sequences and with a FASTQ or a CSV file containing circular consensus sequences of the chimeric library. In addition, the user can also provide one or more FASTQ files of enriched variant libraries after selection rounds of variant expression in the target tissues. *hafoe* returns interactive HTML reports describing the distribution of the parental serotypes in the chimeric libraries, the parental serotype composition and diversity (the *--explore* option), and the sequence content of most successful chimeric variants enriched in the target tissue (the --identify option) (Fig. 1B).

The core of our algorithm for the identification of the composition is the neighbor-aware serotype identification approach that divides each chimeric vector into overlapping windows of defined length (reads), aligns them to parental serotypes, and reduces the options of multiple alignment by the assumption that neighboring windows should have a similar parental origin (Fig. 2).

We calculate the accuracy of our variant composition assessment method by comparing the actual composition labels of the synthetic data with the predictions obtained by *hafoe*. We varied different parameters we set for the identification of variant serotype composition, including the fragment size (read length), the distance between consecutive fragments (step size), and the option we use for filtering out parental serotypes of low quality (filtering parameter). With the best combination of parameters (read length 100 nt, step size 10, and “sum of quality scores” filtering parameter) *hafoe* reached accuracy in the range of 96.3% to 97.5% across the five synthetic datasets (Supplementary Fig. S1).

**Fig. 3:**
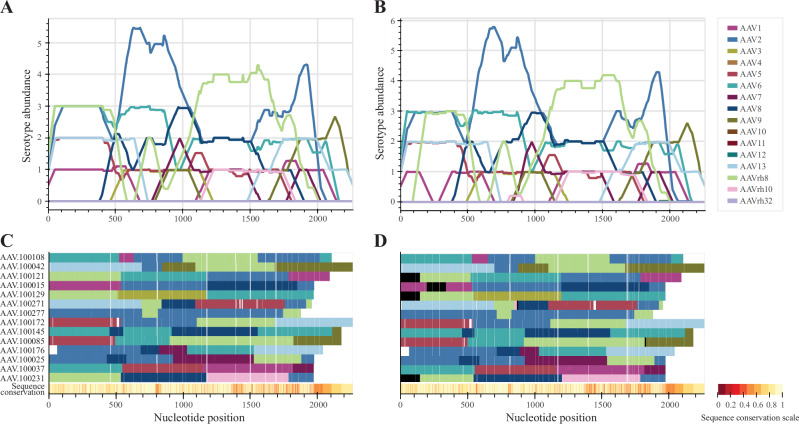
Comparison of true and predicted compositions of chimeric library representative variants in synthetic data. Results for one of the five synthetic datasets are presented here (data for the rest not shown). Position resolved abundance of parental AAV serotypes in the representative variants based on true composition labels stored while generating the data (**A**) and based on the serotype assessment labels obtained by the neighbor-aware serotype identification method of *hafoe* (**B**). The abundances were averaged over 100 nt windows. True (**C**) and predicted (**D**) parental AAV serotype compositions of the representative variants. Multiple sequence alignment (MSA) of the representatives was performed to align the homology regions of the representatives. Gaps in MSA are colored white, unresolved positions are colored black. Conservation levels of the representative variants are displayed in the lower bar of the heatmap, with positions from higher to lower conservation scores represented on a light-to-dark scale.

*hafoe* accurately identified the parental serotype distributions across nearly all the nucleotide positions of the representative variants in the chimeric libraries in the synthetic datasets (Fig. 3A, B). With the default parameters, *hafoe* demonstrated an unresolved position rate of 2.1% and an erroneous identification rate of 1.6%. The inaccurately identified positions were primarily accumulated at the 5’ ends of the variants, specifically positions 1-141 and 193-335 of a few synthetic variants (Fig. 3C, D). This could be explained by the relatively high sequence conservation level of this region in the parental AAV serotypes (Fig. 3C, D, bottom bar).

### Serotype composition of chimeric libraries

**Fig. 4:**
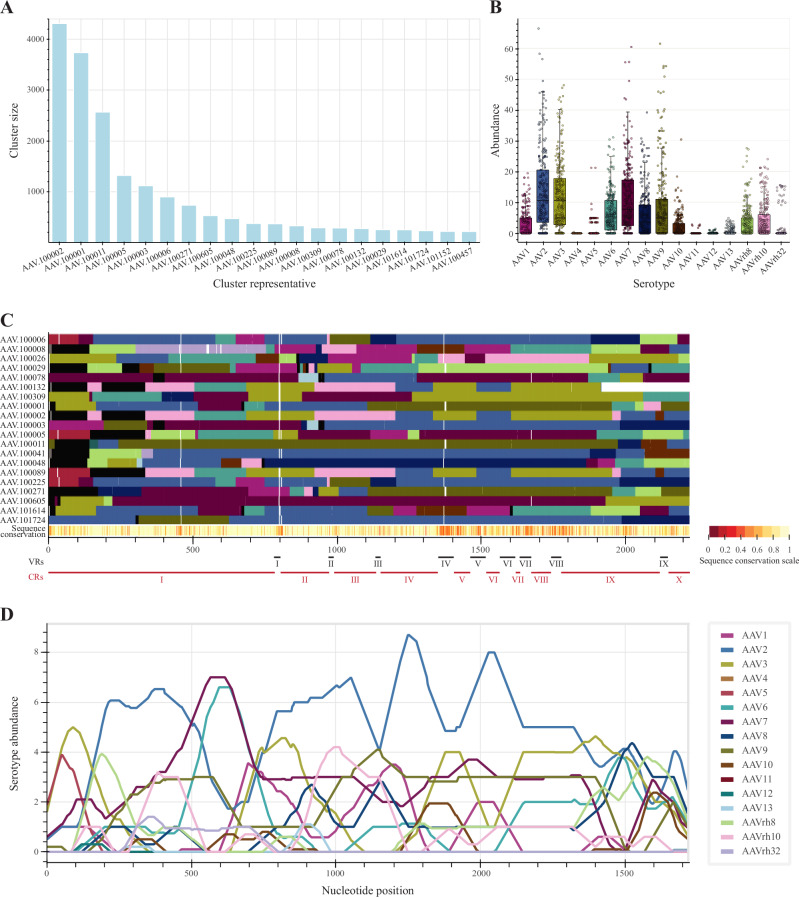
Comprehensive analysis of AAV chimeric library by *hafoe.* **A** Chimeric library cluster size distribution for the top 20 clusters. **B** Parental AAV serotype abundance in the chimeric library. **C** Compositions of the top 20 cluster representatives in terms of parental AAV serotypes. MSA of the representatives was performed to align the homology regions of the representatives. Gaps in MSA are colored white, unresolved positions are colored black, and the positions with no identified serotypes are colored gray. Conservation levels of the representative variants are displayed in the lower bar of the heatmap, with positions from higher to lower conservation scores represented on a light-to-dark scale. The variable regions (VR) I to IX of AAV2 [12] and the remaining conservative regions (CR) are indicated below the heatmap. **D** Position resolved abundance of parental AAV serotypes in the top 20 cluster representatives. The abundances were averaged over 100 nt windows.

**Fig. 5:**
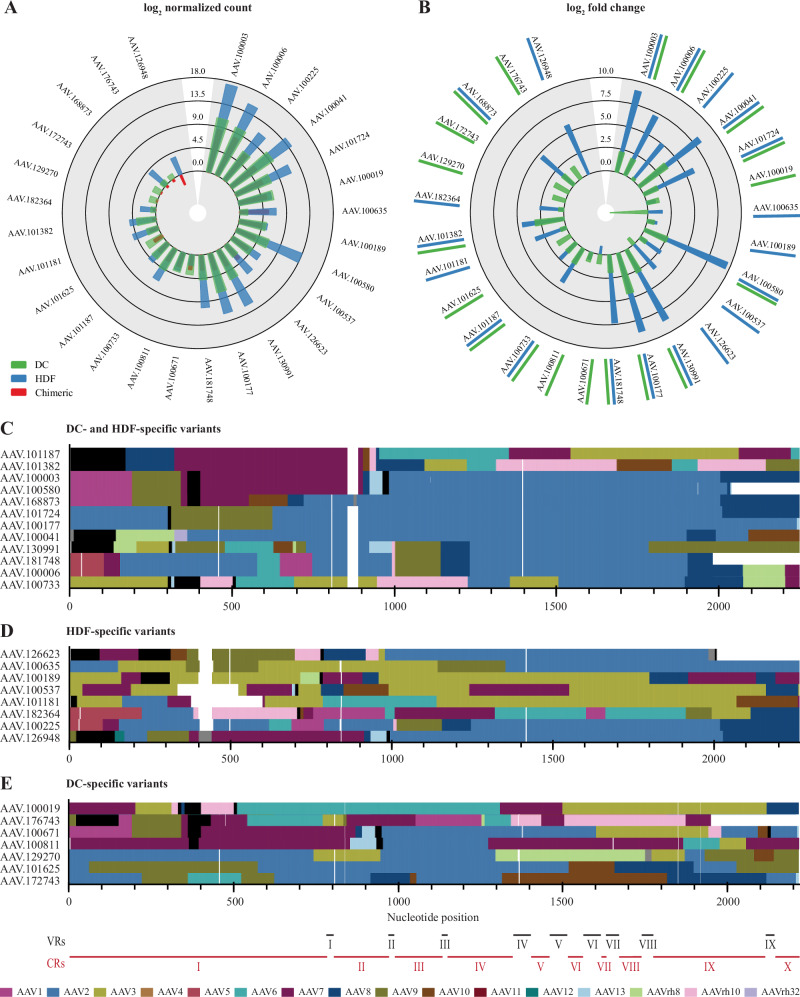
Enrichment profiles of representative variants in HDFs and DCs with a > 1 log_2_ fold change over the chimeric library in either of the cell types. **A** Log_2_ normalized counts of representative variants in chimeric and enriched libraries. **B** Log_2_ fold change of the representative variants in enriched libraries over the chimeric library. The tissue specificity of the variants is color-coded with underlines to their names. **C**–**E** Compositions of the DC- and HDF-specific (**C**) HDF-specific (**D**) and DC-specific (**E**) representative variants in terms of parental AAV serotypes. The variable regions (VR) I to IX of AAV2 [12] and the remaining conservative regions (CR) are indicated below the heatmap.

We demonstrate the application of *hafoe* on two experimental datasets. The first dataset comprises PacBio variant sequence reads generated using an AAV chimeric capsid library, as well as enriched libraries extracted from infected dendritic cells (DCs) and human dermal fibroblasts (HDFs) (Figs. 1A, 4, 5). The second dataset consists of variant sequences from another chimeric library, along with AAV capsid sequences obtained from canine muscle and liver tissues (Supplementary Fig. S2, S3).

The chimeric library sequences were first preprocessed with *hafoe* by sequence length and the open reading frame (ORF) boundaries, leaving 24,111 sequences out of the initial 87,441. These sequences were clustered into 268 groups, each containing up to 4300 variant sequences at 90% identity. The representative sequences of the clusters were used in subsequent analysis (Fig. 4A).

To assess the serotype composition of the capsid variants in the chimeric AAV library, *hafoe* performs in silico fragmentation of the representative sequences and subsequent alignment to parental AAV serotypes, followed by neighbor-aware serotype identification for each base. The variant identification results for both chimeric libraries showed generally high abundance of the AAV2, AAV3, and AAV7 parental serotypes (*p*-value < 0.015, 0.009, 0.045, Tukey honestly significant difference (HSD) post hoc test) (Fig. 4B, Supplementary Fig. S2B). Specifically when examining the top 20 representatives comprising 81.9% of total reads, AAV3, AAV2, and AAV7/AAV6 predominate at nucleotide positions 0–150, 150–500, and 700–1750, and 500–700, respectively (Fig. 4C, D). The AAV4, AAV5, AAV11, and AAV12 were almost not present in the chimeric variants. A similar distribution of serotypes was observed for the second chimeric library in the first 700 bases, however instead of AAV2 in the 700-1750 bp region of the variants we observed dominance of AAV7 and AAV3 (Supplementary Fig. S2 C, D).

### Tissue enrichment

We next sought to use *hafoe* to identify AAV variants with tropism for human dendritic cells (DCs) and dermal fibroblasts (HDFs). For this, we applied our chimeric AAV library on in vitro cultured monocyte-derived DCs and dermal fibroblasts and used *hafoe* to compare normalized abundance counts of representative variants extracted from these target tissues with the abundance of the input chimeric library members. The analysis revealed several capsid variants that exhibited enrichment in DCs and HDFs compared to the input chimeric library (Fig. 5). For instance, 12 variants demonstrated enrichment in both DC and HDF samples, with all of them having a greater log_2_ fold change (logFC) in HDFs. These variants are described by the predominance of AAV7 in the conservative region (CR) I and the variable region (VR) I of the vectors, and AAV2 in the CR/VR II to IX (Fig. 5C). Additionally, 8 variants were discovered to have higher tropism to HDFs alone with a preference for AAV3 in half of them. Finally, 7 AAV library members showed differential enrichment in DCs (Fig. 5D, E), albeit with no observable wild-type AAV preference.

The analysis of canine libraries showed enrichment of AAV9 along the CR I and/or II to IX, including VR I to VIII of the muscle-specific variants, while the liver-specific variants showed preference for AAV7 or AAVrh10 in the region including VR/CR II to VIII (Supplementary Fig. S3).

### Comparison of amino-acid composition of published vectors

**Fig. 6:**
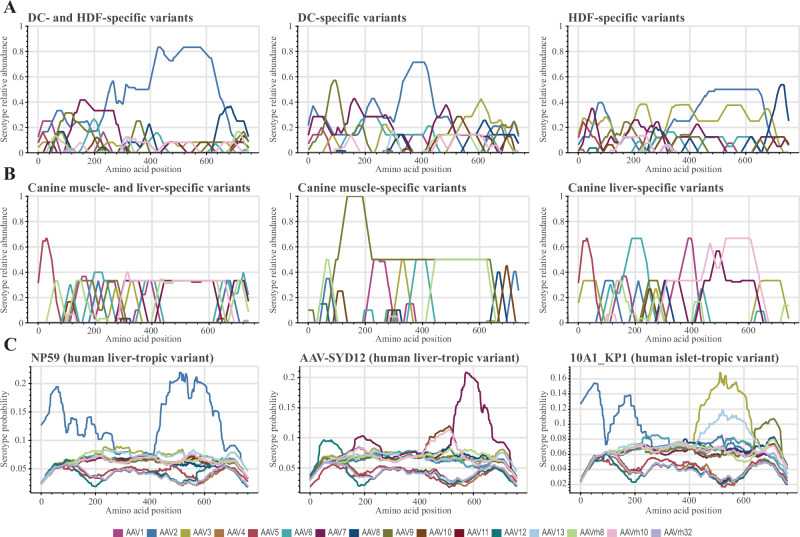
Position resolved relative abundance or probability of parental AAV serotypes in identified tissue-specific variants and previously reported AAV vectors. DC- and/or HDF-specific variants (**A**) and canine muscle- and/or liver-specific variants (**B**) identified by *hafoe*. Human liver-, islet-tropic variants from literature (**C**). The abundances were averaged over 100 nt windows.

To compare our results with previously reported tissue-specific variants, we implemented another feature of analyzing amino acid sequences into *hafoe*. We conducted a comparative analysis involving previously reported AAV vectors NP59 [33], 10A1-KP1 [34], and AAV-SYD12 [14] alongside our tissue-specific variants (Fig. 6).

The NP59 AAV capsid variant represents a human liver-tropic variant generated through DNA shuffling of capsid genes from 10 parental serotypes (AAV1, AAV2, AAV3b, AAV4, AAV5, AAV6, AAV8, AAV9_hu14, AAV avian, and AAV bovine). The NP59 variant has previously been selected through sequential screening in humanized liver mice and against pooled human immunoglobulins due to its enhanced hepatic transduction and immune-evasive properties [33]. AAV2 was predominantly selected in both the N- and C- terminal halves of these variants (Fig. 6C). Another liver-tropic vector, AAV-SYD12, which demonstrates enhanced human hepato-tropism in a liver xenograft mouse model, was created through capsid shuffling of AAV serotypes AAV1-AAV12 [14]. In contrast to the NP59 variant, which was generated in the absence of AAV7 serotype, this one demonstrated a preference for AAV7 in the C-terminus of the capsid, in agreement with what we observe in our canine liver-tropic vectors (Fig. 6B, C and Supplementary Fig. S3). The 10A1-KP1 AAV capsid variant, engineered to improve transduction to primary human islet cells and human embryonic stem cell-derived β cells, was generated through DNA shuffling of capsid sequences from 8 AAV serotypes (AAV1, AAV2, AAV3B, AAV6, AAV8, AAV9hu14, AAV12, AAVrhesus10) and previously generated chimeric AAV-DJ and AAV-LK03 variants [34]. Here we observe the prevalence of AAV2 in the N-terminus, and AAV3 or AAV13 in the C-terminus (Fig. 6C).

## Discussion

Adeno-associated viral vectors are a proven platform for the delivery of therapeutic genes into target tissues with favorable safety profiles and robust expression of transgenes in various tissue types [2, 4]. However, the tropism of naturally occurring AAV vectors does not allow for specific targeted gene delivery to many tissues of interest [1]. In addition, the immunogenicity of AAVs is a concern, as almost half of the human population is seropositive for the common viral capsids [5, 35]. Thus, there is a strong rationale for the development of novel tissue-specific AAV variants as well as bioinformatic tools to analyze their structure and tissue abundance.

Here, we have developed a computational tool *hafoe* for the comprehensive analysis of shuffled DNA libraries. In particular, we demonstrate its use on chimeric AAV variant sequences, produced by recombination-based shuffling of the *cap* gene from several parental AAV serotypes. *hafoe* enables the exploratory analysis of the chimeric AAV libraries revealing the composition of parental serotypes in the new variants and their enrichment in target tissues. Our tool is designed for researchers with no bioinformatics background and runs with a single command line. It produces interactive HTML reports with multiple plots describing the diversity of the chimeric library, the prevalence of parental serotypes, the serotype composition of representative variants, and those enriched in target tissues. In contrast to other tools, *hafoe* isn’t limited by the number of input sequences, can process both DNA and peptide sequences, and can provide output for any number of input libraries. It can also be used for any other type of chimeric libraries, aside from AAVs [21].

We have validated the performance of the neighbor-aware serotype identification algorithm for parental serotype composition assessment implemented in *hafoe* using a synthetic PacBio dataset of in silico shuffled variants. We correctly identify the parental serotypes of more than 96% of the bases in the variant sequences.

As a confirmation of its utility, *hafoe* was implemented to identify novel AAV variants enriched in human dendritic cells (DCs) and dermal fibroblasts (HDFs) as well as in canine liver and muscle tissues. Specifically, *hafoe* was used to characterize two in-house datasets comprising AAV chimeric capsid libraries generated by gene shuffling. *hafoe* was then used to quantify the abundance of variants enriched in studied cells and tissues and determine AAV candidates for subsequent individual evaluation. Upon further validation, variants enriched in DCs pave the way for their potential use in vaccine development, while those abundant in HDFs could be applied to skin gene therapy. Furthermore, *hafoe* provides structural information on characterized AAV variants. For instance, an enrichment for AAV7 and AAVrh10 was observed in canine liver-specific vectors, in agreement with previous results that show that the presence of the variable regions VI to VIII (amino acids 500-700) from AAV7 is advantageous for the liver-tropic phenotype [14].

Overall, *hafoe* provides rich functionality for exploratory analysis of parental AAV serotypes in the newly generated chimeric vectors for researchers with no bioinformatics background. It can also be useful for bioinformaticians as a basis for downstream analysis. For example, one might want to proceed with variant calling to understand whether the presence of the parental serotype itself in the given location of the vector is sufficient to give it its tissue-tropic properties or whether the sequences should also be modified. The researchers might also want to look into the clusters of the representative sequences to find out how variable the sequences can be. By changing the clustering threshold, the users can also perform analysis at higher or lower resolution.

It is important to take into consideration the limitations of this approach. The *cap* gene encodes three structural virion proteins (VP1, VP2, and VP3) through alternative splicing and start codon usage, as well as two other proteins, the phospholipase A2 (PLA2) (in-frame) and the Assembly-Activating Protein (AAP) (alternative frame) [36]. This means that one needs to be careful in interpreting the functional consequences of the observed serotype composition on the final protein products. *hafoe* does not include functionality to predict how the observed variations will specifically affect the translation of these products.

Furthermore, we do not analyze the immunogenicity profiles of the vectors. We have analyzed the variants in this study for their ability to bind HLA receptors to predict their enrichment in the in vivo selected libraries, using NetMHCpan-4.1 and NetMHCIIpan-4.0 tools [37] trained on the IEDB database (https://www.iedb.org/) containing quantitative peptide binding data and the IMGT/HLA database (https://www.ebi.ac.uk/ipd/imgt/hla/index.html) of HLA sequences. However, our results did not show any association between the predicted binding affinities and the selection of the region in the tissue-specific library. Therefore, we did not implement such a feature in *hafoe*. Although recent advances in ML-based structure–function prediction (e.g., AlphaFold [38]) can rapidly generate high-resolution models and functional hypotheses, these in silico predictions still require empirical validation. Directed evolution via DNA shuffling provides complementary, data-driven selection of functional variants, capturing epistatic and context-dependent effects that may not be fully predicted by structure alone.

Finally, we would like to highlight a limitation of our experimental design, which involves a two-step selection process: first, an in vitro screen that enriches for variants with efficient packaging, and second, an in vivo screen that selects for improved transduction. Variants with high transduction potential but suboptimal packaging efficiency may be lost during the initial enrichment step and therefore excluded from subsequent analysis. This potential bias should be taken into account when interpreting the results of such screens.

In summary, we have developed *hafoe*, a user-friendly standalone software designed for the analysis of chimeric AAV datasets, and used it to identify novel AAV variants for human and dog gene delivery. *hafoe* can be used as a tool to inform directed evolution and follow-up rational design approaches for the generation of novel AAV variants with desired tissue tropism [14, 39, 40]. The tool can also be used with other types of shuffled DNA libraries [40–42].

## Supplementary information

Supplementary information

## Data Availability

The data will be made available upon request to authors.

## Code availability

The data will be made available upon publication. The *hafoe* software and code is available for free download at https://github.com/abi-am/hafoe. The repository also contains synthetic data example of chimeric variants at https://github.com/abi-am/hafoe/tree/main/example.
