# Supplementary material for: hafoe: an interactive tool for the analysis of chimeric AAV libraries after random mutagenesis
Source: Gene Ther. 2025 Jul 8;32(5):475–86. doi: 10.1038/s41434-025-00548-3 (PMC12518119; doi:10.1038/s41434-025-00548-3)
Supplement: Supplementary file 1 — Supplementary information [file 41434_2025_548_MOESM1_ESM.docx]

# Supplementary Information


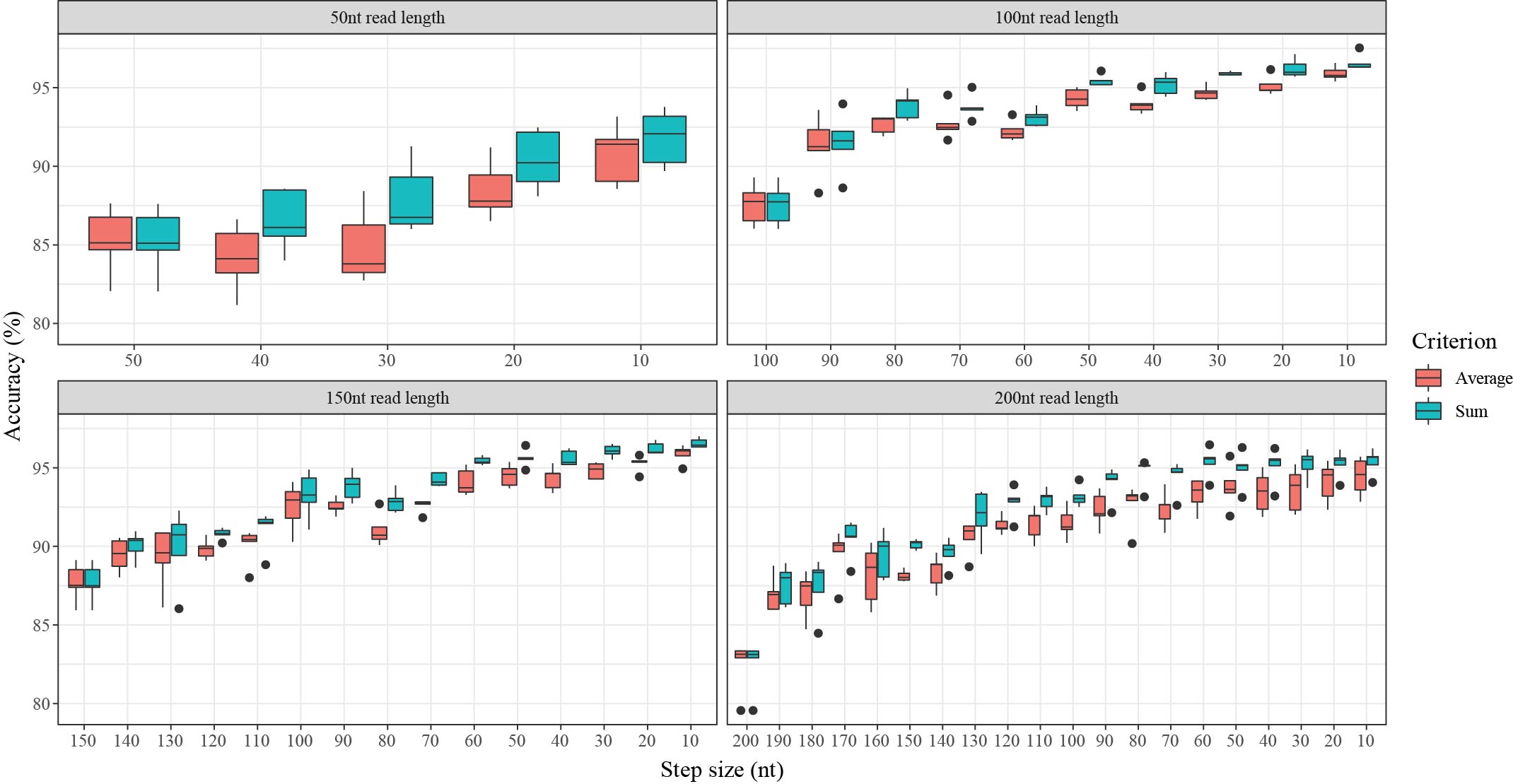


**Supplementary Figure S1: Accuracy of variant composition identification with *hafoe* on synthetic data.** Different combinations of read length (the length of the chopped fragments from variant sequence), step size (the length of the region between the starting positions of two consecutive fragments), and filtering criterion (sum or average of alignment quality scores across all reads mapped to a position per serotype assignment (Figure 1C, Step 1)) input parameters are used to run *hafoe* on five synthetic datasets. Accuracy is computed by dividing the total number of accurately described nucleotides in a sequence by the sequence length, then averaging this value across all the sequences in the dataset. The “sum of quality scores” filtering criterion has, in general, better accuracy compared to the “average of quality scores” filtering criterion. The best combination with read length 100 nt, step size 10, and “sum of quality scores” filtering criterion reached accuracy in the range of 96.3% to 97.5% across the five datasets.


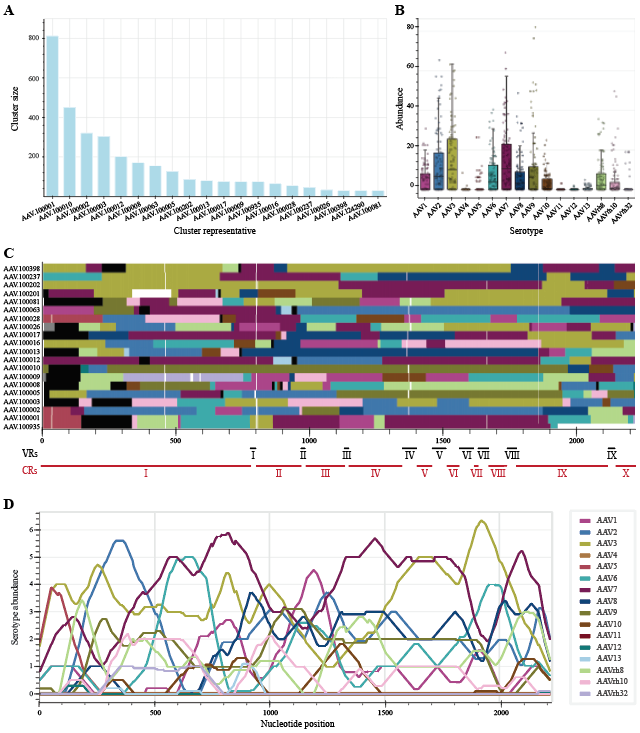


**Supplementary Figure S2: Comprehensive analysis of AAV chimeric library by *hafoe*. A** Chimeric library cluster size distribution for the top 20 clusters. **B,** Parental AAV serotype abundance in the chimeric library. **C,** Compositions of the top 20 cluster representatives in terms of parental AAV serotypes. MSA of the representatives was performed to align the homology regions of the representatives. Gaps in MSA are colored white, unresolved positions are colored black, and the positions with no identified serotypes are colored gray. The variable regions I to IX of AAV2^1^ are indicated above the heatmap. **D,** Position resolved abundance of parental AAV serotypes in the top 20 cluster representatives. The abundances were averaged over 100 nt windows.


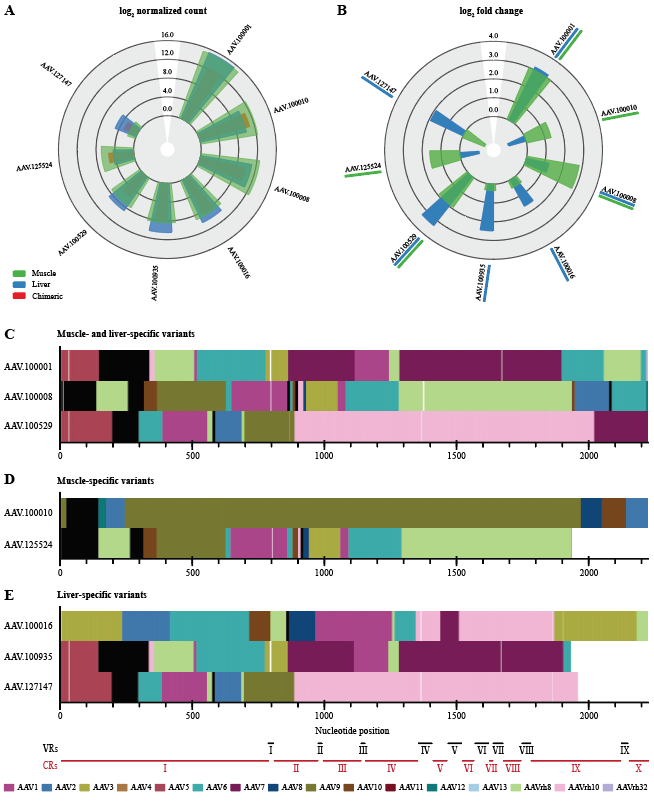


**Supplementary Figure S3: Enrichment profiles of representative variants in dog muscle and liver tissues with a >1 log_2_ fold change over the chimeric library in either of the tissues. A,** Log**_2_** normalized counts of representative variants in chimeric and enriched libraries. **B,** Log**_2_** fold change of representative variants in enriched libraries over the chimeric library**.** The tissue specificity of the variants is color-coded with underlines to their names. **C, D,** and **E.** Compositions of the muscle- and liver-specific (**C**), muscle-specific (**D**), and liver-specific (**E**) representative variants in terms of parental AAV serotypes. The variable regions I to IX of AAV2^1^ are indicated above the heatmaps.

**Supplementary Table S1.** List of primers for reverse transcription of cDNA.

TAAAAGCTTT

AAAGCTTTGA

AGCTTTGATC

CTTTGATCAT

TTGATCATAA

GATCATAATC

TCATAATCAG

ATAATCAGCC

AATCAGCCAT

TCAGCCATAC

AGCCATACCA

CCATACCACT

ATACCACTAG

ACCACTAGTG

CACTAGTGTT

CTAGTGTTAG

AGTGTTAGCT

TGTTAGCTGA

TTAGCTGATC

AGCTGATCTA

CTGATCTAGA

GATCTAGATA

TCTAGATAAC

TAGATAACTG

GATAACTGAT

TAACTGATCA

ACTGATCATA

TGATCATAAT

ATCATAATCA

CATAATCAGC

TAATCAGCCA

ATCAGCCATA

CAGCCATACC

GCCATACCAC

CATACCACAT

TACCACATTT

CCACATTTGT

ACATTTGTAG

ATTTGTAGAG

TTGTAGAGGT

GTAGAGGTTT

AGAGGTTTCT

AGGTTTCTAG

GTTTCTAGTG

TTCTAGTGAG

CTAGTGAGAC

AGTGAGACGA

TGAGACGACT

AGACGACTAG

ACGACTAGTG

# References

1 Drouin LM, Agbandje-Mckenna M. Adeno-associated virus structural biology as a tool in vector development. *Future Virol* 2013; 8: 1183.
